# The Foreign Journals: Surgery

**Published:** 1842-04

**Authors:** 


### SURGERY.

### *Rupture of the Spine, by a violent Muscular Effort.* By M. Lasalle.

A man, thirty-six years old, was taken to the Maison Royale de Santé at Charenton, in a state of furious mania, and it was found necessary to confine him to his chair by means of two leather straps, which went from the upper part of its back and fixed his shoulders, and two others, which went from the same part a little lower down, and were fastened to his arms. After making various efforts to break from his confinement the patient rose up from his seat, threw his head forcibly backwards, and then flung it with great violence forwards. After this last movement the head remained bent on the neck, and the neck on the chest, and his limbs were completely paralysed. The author saw him soon afterwards, and found an absolute loss of power in all the muscles below the neck, except the diaphragm and those of the arms, which were but partially paralysed. There was neither bruise, nor swelling, nor deformity in the course of the vertebral column.

About thirty-six hours after these events, which were witnessed by a keeper and a fellow-patient, the man died, with all the usual signs of pressure on the cervical portion of the cord. On examination, a solution of continuity was found between the fifth and sixth cervical vertebræ; the posterior cervical ligament and the interspinales muscles were ruptured, and no bond of union remained between the spinous processes, whose bases were separated so as to expose the remains of the ligamentum subflavum and the membranes of the cord. The articular surfaces of the superior oblique processes of the sixth vertebra were also exposed, and its left transverse process was fractured. The intervertebral substance was torn; a part remained attached to the fifth, a part to the sixth vertebra; but there was no fracture of the bones, nor any material displacement of them. The dura mater at the seat of the injury was tinged with blood, and the surrounding cellular tissue was infiltrated with it. None of the membranes were torn, but in the interior of the cord, opposite the injury, there was a vast ecchymosis, extending downwards to the second dorsal and upwards to the third cervical vertebra.

[This case affords a new argument in favour of the mild treatment of the insane. *Restraint,* goading the unhappy man to fury, was the undoubted cause of death, and not the insanity merely.]

*Gazette Médicale. Novembre* 27, 1841.

---

### *Wound through the Sternum and the Arch of the Aorta.* By Dr. Casper.

The peculiarity of this unique case is, that the wound was made with a common dinner-knife, only moderately sharp at its point and its edge three and a half inches long, and three fourths of an inch broad. With this a man stabbed his wife up to the hilt. She died in an instant; and the wound was found to have passed right through the upper part of the sternum into the arch of the aorta and the right lung. The bone was cleanly pierced, without any fracture or splintering.

*Casper's Wochenschrift. January* 1, 1842.

---

### *On the Treatment of Ulcers between the Toes.* By Dr. Schlesier.

The author says that an invariably successful method of treating this affection, whether it have a syphilitic origin or not, is to sprinkle them thickly day after day with red precipitate, and then to cover them with dry charpie. The cure is generally effected in a few days.

*Medicinische Zeitung. November* 24, 1841.

*On Syphilitic Muscular Retraction, and its Treatment.*

THIS affection has been only recently observed by M. Ricord. It most frequently affects the flexors of the forearm, at least if a judgment may be formed from several cases that have lately occurred at the Hôpital des Vénériens. The patients had all attained the tertiary state of constitutional syphilis. In the three chiefly noticed, the flexors of the forearm seemed shortened under the influence of a permanent contraction, which prevented the arm from being straightened; but their tissues, though hard and stiff, did not present any appreciable alteration. The muscles were very painful, and the pain was aggravated at night. All the patients were cured in less than six days by the iodide of potassium.

*Bulletin Générale de Thérapeutique. Janvier,* 1842.

---

*On the means of arresting the Hemorrhage produced by certain fibrous Polypi of the Uterus.* By M. LISFRANC.

WHEN local astringents and internal medicines fail, and the patient is too much reduced to permit the removal of the polypus, M. Lisfranc, having observed that the source of bleeding is not in the tumour itself, but in the membrane investing it, adopts the following method. If the polypus is low down, he seizes the membrane with his finger and thumb, and pulls it off; "in a word, he peels the polypus." The hemorrhage is checked, and does not return "after this little operation." If the polypus be too high to be thus treated, he seizes it with hooks, and through a large speculum, after stopping the temporary bleeding by cold and astringents, he sponges its whole surface with the acid proto-nitrate of mercury "on the instant the hemorrhage stops." By either of these means time may be gained for the patient to recover her strength, and be prepared for the operation of removing the tumour.

*Bulletin Générale de Thérapeutique. Janvier,* 1842.

---

*On Iodine Injections in the Treatment of Serous Cysts.* By M. VELPEAU.

M. VELPEAU was induced, by the success which attended the use of this means in the treatment of hydrocele, to adopt it for various kinds of serous cysts, enlarged bursæ, &c., about the knee, in the axilla, breast, neck, and other parts. The proceeding consists in puncturing the cyst with a trocar proportioned to its size, emptying it, and then injecting through the canula a mixture of one part of tincture of iodine with two parts of water. This having been retained for a few seconds, should then be nearly all drawn off again. The pain produced by this operation is generally inconsiderable, and ceases after about a quarter of an hour; a day or two after reaction takes place the cyst inflames, though never severely; in two or three days more, resolution commences, the cyst diminishes, grows pale, shrivels, and in two, four, or six weeks the cure is perfected. It is rarely delayed beyond this time, but it may be accelerated by rubbing the skin over the cyst with mercurial or iodine ointment when all the signs of acute inflammation in it have passed away.

*Bulletin Général de Thérapeutique. Novembre,* 1841.

